# A Systematic Review of Internet-Based Interventions for the Prevention and Self-Management of Cardiovascular Diseases among People of African Descent

**DOI:** 10.3390/ijerph19148872

**Published:** 2022-07-21

**Authors:** Jesse Enebi Usman, Charmaine Childs, David Rogerson, Markos Klonizakis

**Affiliations:** 1 Lifestyle Exercise and Nutrition Improvement (LENI) Research Group, Department of Nursing and Midwifery, Sheffield Hallam University, Sheffield S10 2BP, UK; 2 College of Health, Wellbeing and Lifesciences, Sheffield Hallam University, Sheffield S10 2BP, UK; 3 Sports and Physical Activity Research Centre, Sheffield Hallam University, Sheffield S10 2BP, UK

**Keywords:** cardiovascular diseases (CVDs), digital health, mHealth, digital technology, people of African descent (PAD), internet-based intervention (IbIs), behavioural change, lifestyle interventions, cardiovascular risk factors

## Abstract

Cardiovascular disease (CVD) risk factors, morbidity, and mortality among people of African descent (PAD) appear to be higher than in the general population. While it has been found that lifestyle changes can prevent around 90% of CVDs, implementing an effective lifestyle programme is expensive and time-consuming. It has been demonstrated that Internet-based interventions (IbIs) can effectively and inexpensively encourage lifestyle modifications to prevent and manage chronic diseases. Although a number of studies have examined the effectiveness of IbIs in the general population, no comprehensive study of the usefulness and acceptability of IbIs among PAD has been conducted. This is the knowledge gap that this study aimed to address. We searched MEDLINE, EMBASE, CINAHL, and Web of Science to identify eligible studies published from inception to February 2022. Thirteen articles met our criteria for inclusion. Our textual narrative synthesis produced inconsistent results; nonetheless, high acceptability of IbIs and a considerable improvement in clinical and behavioural outcomes associated with CVDs were reported in several trials. The findings of this review are constrained by clinical, methodological and statistical variability among the studies. To have a good grasp on the effect of IbIs on behaviour change in PAD at risk of CVDs, large-scale longitudinal studies with long-term follow-up are required.

## 1. Introduction

Cardiovascular diseases (CVDs) are a broad term for various conditions affecting the heart or blood vessels. These disorders include stroke, heart failure, aortic disease, myocardial infarction, peripheral arterial disease, and coronary artery illnesses such as angina pectoris [1]. Diabetes, hypertension, poor diet, smoking, obesity, high blood cholesterol, physical inactivity, and excessive alcohol intake are all cardiovascular risk factors [2,3]. Globally, CVDs are the leading cause of death, accounting for 17.9 million deaths in 2015 [4]. These diseases are responsible for one in every four deaths in the United Kingdom (UK) [5]. CVDs affect about 7.6 million people in the UK, and management costs the economy over £15 billion yearly [6]. Up to 90% of CVDs are preventable through lifestyle changes involving healthy eating, exercise, sleep hygiene, limiting alcohol consumption, and avoidance of tobacco [1]. As outlined in the National Health Service (NHS) long-term plan, priorities are placed on preventing CVDs and preventative health technologies (digitally enabled care) [7]. Accordingly, developing and testing innovative and scalable CVD prevention strategies are essential.

CVDs exhibit significant ethnic or racial disparities in prevalence, risk factors, morbidity, and associated mortality [8,9]. Studies have found that people of African descent (PAD) experience excess CVD-related mortality and morbidity compared to the general population [10,11], with lifestyle, cultural and socioeconomic factors potentially playing crucial roles in this phenomenon [3,12,13]. It has also been reported that the high prevalence of CVDs in PAD may be due to poor engagement with services that promote self-management. Cultural barriers such as health illiteracy, lack of trust in conventional medicine, and absence of culturally sensitive advice hinder engagement [14]. Furthermore, physiological factors such as decreased plasma renin activity, excessive salt sensitivity, and lower levels of endothelium-derived vasodilators have been linked with higher risks of CVDs morbidities and mortalities in PAD worldwide [15,16,17]. CVDs and health outcomes in PAD may have been worsened by the additional strain brought on by the coronavirus disease (COVID-19) pandemic and related stressors [18,19]. Consequently, novel approaches for CVD prevention in PAD are required.

Self-management encourages individuals to take control of their health [20]. Adherence to a balanced diet, quitting smoking, drinking in moderation, or increasing physical activity can be encouraged through CVD self-management practices [21,22,23]. Some studies have shown that Internet-based interventions (IbIs) can promote self-management and prevention of CVDs and risk factors [24,25,26]. These innovative health care technologies create a potential for scalability at low cost and can be adapted to meet the needs of individuals with different medical conditions [27]. According to recent data, the Internet has emerged as a key source of information for individuals of all ages; its use among adults in the UK is currently about 92%, according to recent data [28]. The Internet can potentially become an effective instrument for delivering healthcare interventions on a large scale to promote self-management and the prevention of chronic diseases [29]. Face-to-face interventions are equally effective, but these may be expensive to facilitate [30].

The purpose of this review was to identify knowledge gaps by systematically synthesising the current literature on the effectiveness and acceptability of IbIs for the prevention and self-management of CVDs in PAD. The findings of this review can be used as a basis for consultation with service providers, patients, carers, and minority populations, which may lead to service improvements and the development of collaborative ideas.

## 2. Materials and Methods

This review adhered to the Preferred Reporting Items for Systematic Reviews and Meta-Analyses (PRISMA) criteria [31], and the review protocol was registered with PROSPERO (ID: CRD42022316357). IbIs were defined in this review as customised, participant-centred preventative programmes or interventions provided via the Internet. We examined peer-reviewed and grey literature for IbIs culturally tailored towards the prevention and self-management of CVDs in adults of African ancestry. Primary outcomes of interest include incident CVDs, cardiovascular mortality, and changes in cardiovascular risk factors such as high blood pressure (BP); level of physical activity; glycated haemoglobin A1c (HbA1c); weight; low-density lipoprotein (LDL) cholesterol; smoking status; or a composite cardiovascular risk score.

### 2.1. Search Strategy

J.U. developed the search strategy with help from a specialist librarian. The strategy was structured according to patient characteristics, type of intervention, comparison, outcomes, and study design (PICOS) and reviewed by C.C., D.R., and M.K. (Table 1). The literature search was undertaken in two stages. J.E.U. undertook a preliminary literature scan in MEDLINE and CINAHL to identify keywords and relevant subject headings improved and used in the second literature search. The second search, a more thorough search than the first, was conducted by J.E.U. in MEDLINE (EBSCOhost), Web of Science, EMBASE, and CINAHL (EBSCOhost) from inception to February 2022. J.E.U. thereafter searched the reference lists of eligible publications manually to identify studies that were not captured during the comprehensive search. J.E.U. also searched for relevant grey literature in ProQuest, Grey Literature Report, OpenGrey, and clinical trial registers (UK Clinical Trial Register, EU Clinical Trial Register). Due to a lack of translation resources, only papers published in English were considered eligible. There were no limits based on the year of publication.

### 2.2. Study Selection

The literature search results were reviewed over two phases according to predetermined inclusion criteria based on PICOS (Table 2). The search results were exported to EndNote (a web-based reference manager) to remove duplicates. J.E.U. independently screened the titles and abstracts of studies retrieved to identify eligible studies to be considered in the next phase. In the second phase, the full texts of the potentially eligible studies were assessed independently by J.E.U., D.R., and M.K. for inclusion in the study. Any disagreement or disparities were settled by discussion.

### 2.3. Data Extraction and Analyses

A data extraction form was developed by J.E.U. using Microsoft Excel software [32] to extract relevant data from the eligible studies. J.E.U. critically reviewed articles that met the inclusion criteria to extract descriptive data, including the year of publication, study location, study setting, population studied, study aim, characteristics of intervention and control conditions, type of intervention, methodology applied, and main findings. Before implementing the data extraction form, D.R. reviewed it and pre-tested it with J.E.U. to ensure relevant information was captured. J.E.U. conducted data extraction, C.C. and M.K. subsequently reviewed this to ensure accuracy and consistency; disagreements were settled by discussion. This review provides a narrative summary of the findings from the studies that were included.

A meta-analysis was not undertaken due to the clinical, methodological, and statistical heterogeneity among individual studies. High heterogeneity arising from clinical, methodological, or differences in outcomes assessments suggests that studies do not all report the same quantity; therefore, combining these highly heterogeneous studies can yield meaningless results [33]. Alternatively, we used narrative synthesis to summarize the findings from individual studies. Narrative synthesis is a standard alternative method for quantitative data evaluations where statistical synthesis is not practicable [34]. This synthesis method aggregates studies into homogenous groups to develop structured summaries, and it can be useful in synthesising both qualitative and quantitative data [35,36].

Although meta-analysis is useful for generating meaningful conclusions from data and can help eliminate interpretation errors, there are circumstances in which it can be more detrimental than beneficial. According to Section 10 of Chapter 10 of the Cochrane Handbook for Systematic Reviews of Interventions, version 6.3 [33], a meta-analysis may be meaningless if trials differ clinically because true differences in effects may be concealed. Moreover, if bias exists in some individual studies, meta-analysis can compound the flaws and yield a misleading result that may be viewed as having greater credibility [37]. Therefore, the decisions on what should and should not be combined are necessarily subjective; they require debate and clinical discretion [38].

In cases when it is considered that the studies being pooled are heterogeneous, random-effect models may be utilised as a statistical model even when a test for heterogeneity does not yield a significant result. However, random effect models can be hindered by a high percentage of studies’ non-negligible variability, making these constraints relevant in practice [39]. Furthermore, within-study and between-study variances prevalent in this review are sources of error in a random-effects model [40].

### 2.4. Risk of Bias Assessment

J.U. assessed and evaluated each selected study’s quality and risks of bias using the Jadad score [41] and the Cochrane Collaboration risk assessment tool [33,42]. The studies’ quality and risks of bias were assessed by gathering information regarding blinding (outcome assessor and participant), allocation concealment, number of randomised participants, reporting pre-specified outcomes, number of participants excluded, attrition, and power calculations. D.R. further reviewed the results of the risk of bias assessment, and disagreements were settled by discussions.

## 3. Results

**Figure 1:**
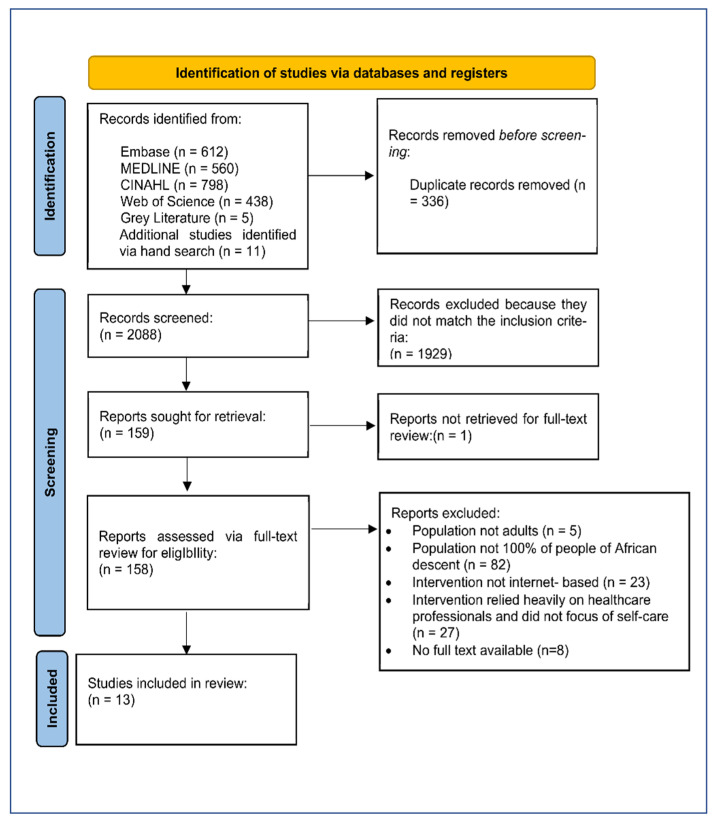
Flow Diagram of Included Studies.

Figure 1 indicates the screening and selection of articles. The electronic search yielded 2413 results, 336 of which were duplicates. Eleven new articles were added via a manual search. One thousand nine hundred and twenty-nine of the remaining 2088 articles did not match the inclusion requirements. One study could not be retrieved for full-text review. The full-text screening was conducted on the remaining 158 studies. Following the full-text review, 145 articles were eliminated for the reasons shown in Figure 1. This review covered 13 articles providing analysable CVD outcome data.

### 3.1. Study Characteristics

The characteristics of the studies reviewed are summarised in Table 3. All the studies were conducted in the United States of America (USA). Two studies were predominantly concerned with CVD prevention [43,44]. The remaining 11 studies were mostly concerned with preventing or self-management of CVD risk factors, including hypertension, diabetes, overweight and obesity, physical inactivity, and smoking [45,46,47,48,49,50,51,52,53,54,55]. One study explored the use of IbIs in the prevention and self-management of hypertension and diabetes [52]. A total of 1203 Black adults participated in the studies, with 527 randomised to IbIs and 676 to control groups. Sample sizes ranged from 20 to 337 participants, and the mean age of participants was 50 years. Six studies utilised a female-only sampling frame [45,47,48,49,53,54], and the remaining seven focused predominantly on females (82.4%). Socioeconomic status, regional information, and prior internet usage were not reported universally. Eleven of the studies were RCTs [43,44,46,47,48,50,51,52,53,54,55] and two were pre-test and post-test quasi-experiments [45,49]. The duration of follow-up in randomised controlled trials and quasi-experimental studies varied between 30 days and 12 months. The usual duration of follow-up was one, three, or six months. Most studies were published during the last decade (85.7%). Participants were recruited from metropolitan areas [44,45,46,48,51,55], big academic medical facilities [49], primary care and outpatient clinics [52,54], churches [43,47], and an online community [50,53].

### 3.2. Intervention Modalities and Features

Seven studies were based on health promotion theories, while four were evidence-based but not based on any specific theory or model. The theories adopted are the Precaution Adoption Process Model [43], Social Cognitive Theory [48,49,50,51,53], Motivational Interviewing [50], and Information-Motivation Behaviour Skills model of adherence [52]. Migneault et al. [50] utilised social cognitive theory, motivational interviewing, and transtheoretical models to design their intervention. The mobile health modalities used in the studies are social media [45,48], smartphone applications [43,44,52,55], voice technologies [50,54], online videos [47], and websites [46,49,51,53,55]. The intervention in one of the studies was in the form of website and smartphone applications [55]. Given the emphasis on promoting self-management, some studies required participants to use wearable technologies such as sensor-enabled devices, wireless or Bluetooth-enabled scales, and pedometers [45,46,48,53]. The IbIs in the studies reviewed feature activity tracking, communication aid, peer support, and reward-based motivation such as goal setting.

### 3.3. Comparison

Eight studies examined the effectiveness of various IbIs compared to usual care [43,46,47,48,50,52,53,54]. Two studies compared the outcomes of pre- and post-intervention [42,46]. One three-arm study compared the outcomes of interventions delivered via tailored Internet, tailored print, and standard Internet [51]; another study compared two approaches to engagement with a behavioural intervention technology for CVDs [44]. Finally, one study contrasted a tailored website with standard text message intervention [55].

### 3.4. Risk of Bias and Quality Assessment

**Figure 2:**
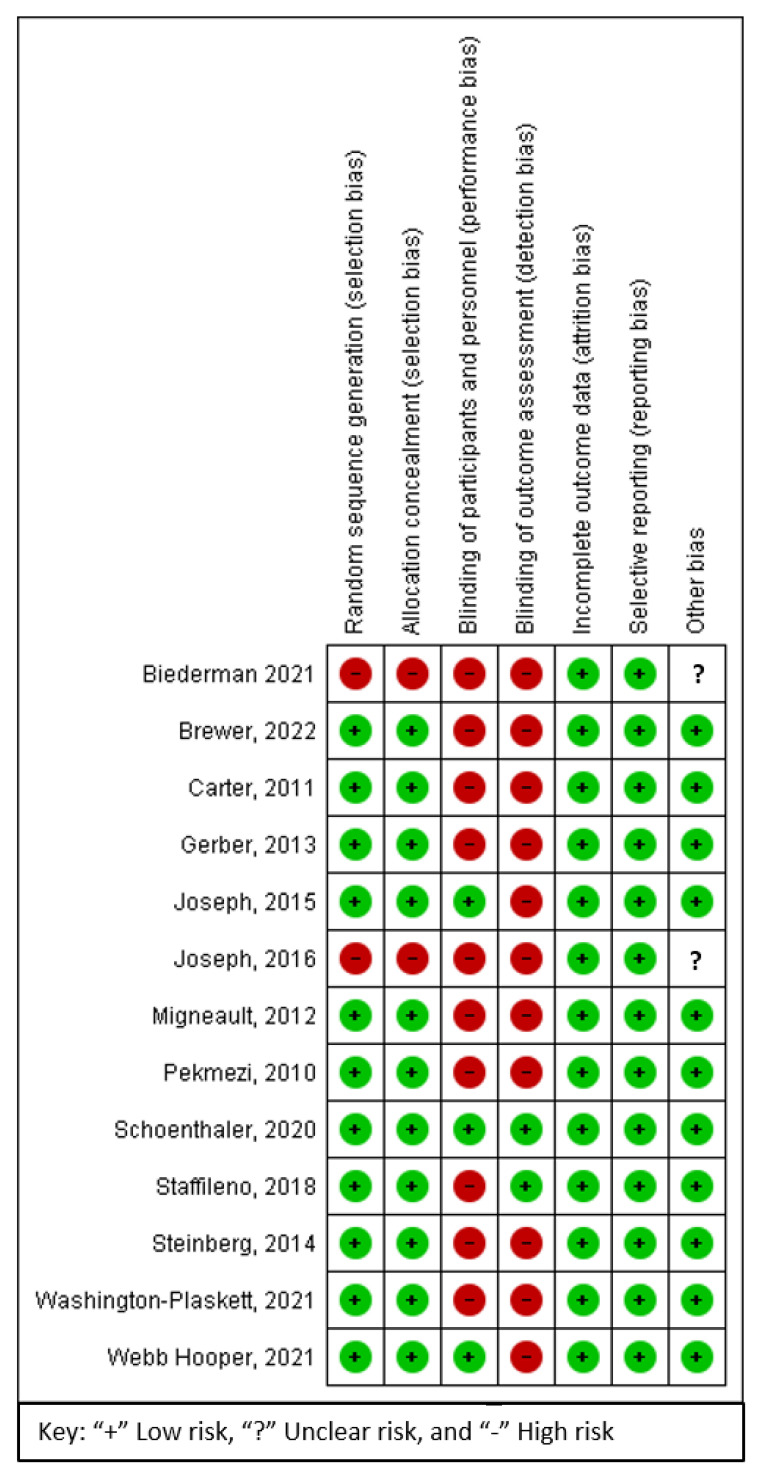
Summary of Reviewers’ Assessments of Each Study’s Risk of Bias [43,44,45,46,47,48,49,50,51,52,53,54,55].

**Figure 3:**
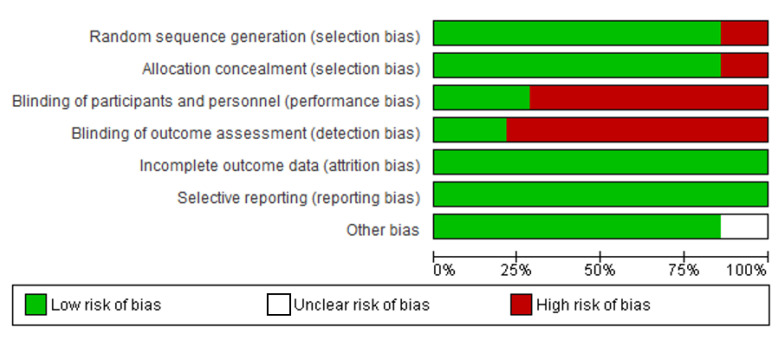
Graph of Reviewers’ Assessments of Each Study’s Risk of Bias.

Figure 2 and Figure 3 present a summary and graphical representation of the studies’ risk of bias. Five of the 13 papers considered in this review exhibited a minimal risk of selection bias. Because of the nature of mHealth interventions, participant blinding was not possible. Therefore, single blinding of outcome assessors was used to evaluate detection bias. In terms of quality, the Jadad Scale (also known as the Oxford quality scoring system) was used to access the studies.

While no study was entirely free of bias, four of the RCTs were ranked ‘high’ (Jadad score of 4/5) because they reported blinding; three studies blinded participants to different intervention arms and permutations [48,52,55], and one study blinded investigators to intervention permutations [53]. Seven RCTs were also ranked ‘high’; however, they had a lower Jadad score (3/5), as they did not provide any information regarding blinding [43,46,47,50,51,54,55]. The remaining two studies were ranked ‘low’ (Jadad score of 2/5) because there were quasi-experiments and did not provide information regarding blinding [45,49].

Six studies reported power calculations [43,44,49,50,52,55]. All the studies had a low risk of attrition bias due to the low withdrawal rate or loss of follow-up. Three trials employed the intention to treat analysis [47,50,52].

### 3.5. Clinical Outcomes

Carter et al. [46] found that IbIs significantly reduced HbA1c levels; however, Schoenthaler et al. [52] reported that there was no significant difference in HbA1c and Diastolic Blood Pressure (DBP) levels prior to and post-intervention. One study found no difference in systolic and diastolic blood pressure between control and treatment groups [50], while Schoenthaler et al. [52] found an improvement in Systolic Blood Pressure (SBP) between groups (4.76 mm Hg; *p* = 0.04). Two trials evaluated the effect of IbIs on anthropometric measurements; one of the studies observed a considerable weight loss during the intervention [54], but Gerber et al. [47] found no significant weight differences between groups. Life’s Simple 7 (LS7) was the focus of two studies that reported CVD outcomes: Brewer et al. [43] reported no changes in LS7 after the intervention; however, Washington-Plaskett et al. [44] reported significant improvements in LS7.

### 3.6. Behavioural/Lifestyle Outcomes

Physical activity (PA) increased in eight studies [43,44,45,48,49,50,51,53]. Biederman et al. [45] reported a significant increase in weekly steps by as much as 190%, and nearly 80% of participants became active at least two days a week after the intervention (35.7%). On the other hand, Gerber et al. [47] reported no significant changes in PA post-intervention. IbIs that promote CVD self-management were successful in Washington-Plaskett et al. [44] and Brewer et al. [43].

The two studies that aimed to improve dietary quality using IbIs produced mixed results. In one study [50], the intervention was associated with an increase in the overall diet quality score (+3.5 points, *p* < 0.03), but Gerber et al. [47] did not find any significant changes in dietary quality between the intervention and control groups. Additionally, Migneault et al. [50] also reported an improvement in energy expenditure (+80 kcal/day, *p* < 0.03) between the intervention and control groups.

### 3.7. Acceptability and Adherence

Seven studies reported on the acceptability of the interventions by the participants. Acceptability was significantly high in all seven studies [43,48,49,50,51,52,55]. According to Migneault et al. [50], participants with lower levels of education perceived the IbIs to be more enjoyable, helpful, useful, practical and enlightening (r = 0.19–0.29, *p* = 0.002–0.02).

High adherence to the intervention was reported by five studies [44,46,48,52,54]. Adherence to recommended exercise sessions was low in Joseph et al. [48]. Schoenthaler et al. [52] reported a significant improvement in medication adherence between the intervention and control groups. In Migneault et al. [50], an intent-to-treat analysis revealed that the treatment group’s adjusted 7-item Morisky Medication Adherence Scale scores improved by 0.19 points compared to the control group. However, this difference was not statistically significant (*p* = 0.25).

Gerber et al. [47] found no significant difference in self-efficacy and social support during the maintenance period of their intervention. No serious adverse event was reported in any study. The outcomes of the interventions and the quality ratings of the studies reviewed are summarised in Table 4.

## 4. Discussion

To the best of our knowledge, this is the first review that looked explicitly at IbIs for the prevention and self-management of CVDs in PAD. We found 13 studies that fulfilled our inclusion criteria, and all the studies were conducted in the USA and published between 2010 and 2022. Two studies focused on preventing CVDs using IbIs; the remaining 11 studies targeted the prevention and self-management of CVD risk factors. The outcomes targeted were: LS7, medication adherence, hypertension, diabetes, physical inactivity, dietary quality, smoke cessation, medication adherence, obesity, and overweight. Meta-analysis was not conducted because of clinical, methodological and statistical heterogeneity; nevertheless, our findings show that IbIs may influence behaviour as part of CVD prevention and self-care.

Results between studies varied: for example, Schoenthaler et al. [52] observed no significant difference in HbA1c levels prior to and post-intervention; however, Carter et al. [46] found that IbIs significantly lowered HbA1c. Also, Schoenthaler et al. [52] observed an improvement in SBP (4.76 mm Hg; *p* = 0.04) across groups, while one study found no difference between control and treatment groups in this parameter [50]. On anthropometric parameters, there was a considerable weight loss after the IbI in one trial [54], but Gerber et al. [47] reported no significant variations in weight between the groups. Washington-Plaskett et al. [44] found considerable improvements in LS7 after the intervention, while changes prior to and post-intervention in Brewer et al. [43] were not considered significant. It is still unclear which mHealth interventions are most effective, even though websites and mobile phone applications have shown the most promising results in PAD in this review. No apparent correlations were observed between the effectiveness of the interventions and follow-up periods or the behaviour change approach used. Due to the small number of studies reviewed and inconsistent results, it is difficult to deduce the effectiveness of IbIs on CVD outcomes and risk factors. Further studies are required before a firm conclusion can be drawn on the impact of IbIs’ on CVD risk reduction in PAD.

All the studies considered in this review showed that IbIs were well-accepted by the intended users. It is important to note that all participants in the studies reviewed were required to be Internet literate; therefore, more studies are required to determine whether IbIs have the same effect on individuals with limited or no Internet access or literacy. There is a growing fear that mHealth will unintentionally exacerbate health disparities if some people have limited access to or familiarity with technology [56]. None of the studies examined whether IbIs were cost-effective, despite the widespread belief that this is an advantage of mobile health technology (mHealth). Particularly essential in cases where patients require at-home monitoring equipment or where interventions necessitate mobile-broadband data subscriptions, cost-effectiveness varies significantly from country to country [57].

Even if it was not stated explicitly, each intervention in this review utilised some form of behaviour change theory (BCT). Hall et al. [58] found that the most successful mHealth interventions were theory-based and provided personalised, individualised, and bidirectional messaging. However, we discovered in this review that the degree of intervention tailoring had no effect on outcomes and that increasing the number of BCTs did not result in the higher behavioural change [59,60]. As a result of the small number of studies included (with a total sample size of slightly more than 1200 participants) and the heterogeneity in the design and reporting of intervention characteristics, we were unable to draw definitive conclusions about the characteristics of effective IbIs in this review [56,57,58]. Before making valid claims about the effectiveness of an intervention, rigorous trial designs with correct power are required [58,61].

### 4.1. Strengths and Limitations of the Studies Reviewed

One strength of this review is that all the studies included are either RCTs or quasi-experiments; however, only six studies included power calculations for behavioural outcomes [43,44,49,50,52,55]. Power is the likelihood of correctly rejecting the null hypothesis that the sample estimates in the underlying population are not statistically different between study groups [62]. The calculation of power and sample size is critical in clinical research, and the best study has a high level of power of at least 80% [63].

All randomised participants were accounted for, and three studies described using intention to treat analysis [47,50,52] Randomisation reduces the chance of a difference in prognosis across groups but does not prevent biased outcomes assessment. This makes blinding an essential methodological component of RCTs [64]. Another limitation of this study is that only four RCTs were blinded, and the two quasi-experiments ranked ‘low’ on the Jadad scale. Only six studies had their trials registered [43,48,50,52,54,55]. A few studies included in this review were either unregistered or did not provide protocol information. According to Moher et al. [65], it is generally established that studies with unclear methodological reporting tend to overestimate treatment effects.

All the studies were conducted in the USA. Similar studies are needed in other countries, especially in low- and middle-income nations with a high prevalence of CVDs and insufficient secondary preventive tools [66]. Additionally, research of this nature is needed in developed countries such as the UK, because of the presence of a large population of PAD.

There is evidence that a diet high in sodium can raise blood pressure, which is a key risk factor for CVDs, as well as several other health problems prevalent among PAD [67,68,69]. The World Health Organization (WHO) recommends that countries design and implement national salt reduction plans so that people can meet the recommended salt intake level of 5 grammes per day [68]. Although a reduction in the amount of salt consumed in the diet may significantly influence the prevention of CVDs in this population, none of the interventions we evaluated in this review targeted dietary sodium.

### 4.2. Limitations of the Review

This review included only English-language articles without date limits. Although some studies might have been missed during our search, we made every attempt to minimise this risk by pre-testing our search parameters and reviewing relevant reference lists thoroughly. A meta-analysis could not be performed because of the wide variation in outcome data and heterogeneity of the literature identified. This means that statistical analyses of study data could not be performed; however, we were able to evaluate the quality of the evidence and highlight both trends and gaps within the evidence qualitatively.

### 4.3. Important Findings and Recommendations

- Even though the results of this review were mixed, there is potential for IbIs to improve clinical and behavioural outcomes of CVDs.
- Despite the compelling evidence supporting the critical role sodium plays in the regulation of blood pressure, which is central in the development of CVDs, none of the studies reviewed in this study focused on dietary salt reduction. This knowledge gap thus provides an opportunity for future research.
- Most of the studies were conducted in the past five years; this suggests a recent emphasis on IbIs in the prevention and promotion of self-management of chronic diseases. Although IbIs were gaining popularity before the COVID-19 pandemic, the demand for these innovative technologies has surged since the outbreak.
- There is a need for robust research designs and long-term follow-ups to determine whether IbIs can permanently cause lifestyle changes in PAD at risk of CVDs.
- All the studies were carried out in the United States. Similar studies are required in other countries with a substantial PAD population.
- Although cost-effectiveness is frequently cited as an advantage of mHealth, none of the studies we reviewed examined the cost-effectiveness of IbIs.

## 5. Conclusions

IbIs has the potential to close the gap in cardiovascular health disparities. Our review looked at 13 studies that used IbIs to deliver behaviour change interventions to prevent or promote self-management of CVDs in PAD. The outcomes of the studies reviewed are inconsistent; nonetheless, many of the studies reported significant improvements in clinical and behavioural outcomes of CVDs in PAD. All the studies were conducted in the USA, hence the need for similar studies to be conducted in other countries. To gain a clearer knowledge of the effects of IbIs on the prevention and self-management of CVDs in the PAD, large-scale, longitudinal studies are required.

## Figures and Tables

**Table 1:** Search Strategy as Defined by the PICOS Strategy.

PICO Terms	Search Term(s)	Search Strategy
Population	Adults of African Descent with CVDs or risk factors	“People of African descent” OR “Black British” OR “Black*” OR “Black African*” OR “Black Caribbean*” OR “Afro-Caribbean*” OR “African American*” OR “Black Ethnic Group”
Intervention	Interventions delivered via the Internet	“Mobile Technolog*” OR “Lifestyle intervention*” OR “Lifestyle” OR “Health Technolog*” OR “Internet” OR “Online” OR “Web-Based” OR “Digital Health” OR “Internet-Based Intervention” OR “Telemedicine”
Comparison	Interventions not delivered via the Internet	
Outcome	Changes in behaviour and Individual CVD risk factors. Adherence and acceptability	“Cardiovascular risk factors” OR “Weight Management” OR “Cardiovascular Disease*” OR “Cardiometabolic” OR “Body Mass index” OR “Waist Circumference” OR “Blood Pressure” OR “Haemoglobin A1c” OR “Fasting Plasma Glucose” OR Triglyceride OR “Total Cholesterol” OR LDL-C OR HDL-C OR “Physical Activit*” OR “Medication Adherence” OR “Smoking Cessation”
Study design	Experimental or quasi-experimental	

**Table 2:** Eligibility Criteria Based on PICOS Strategy.

Population: Adults (≥18 years) of black African ancestry PAD with at least one CVDs or CVD risk factor
Intervention: The interventions must be delivered through the Internet The study duration was at least four weeks, and follow-ups were at least three or more months Intervention is culturally relevant to people of African ancestry Only studies published in English are included
Comparator: Interventions are not delivered via the Internet Addressed self-care of at least one CVDs or risk factor
Outcomes: Changes in behaviour and Individual CVD risk factors Adherence and acceptability
Study Design: Experimental or quasi-experimental

**Table 3:** Summary of Study Characteristics.

Study	Setting	Design/Duration/Intervention Type	Demographics Information and Study Characteristics					
			N	Dropout	Gender [%]	Ethnicity [%]	Mean Age [Years]	Target Disease
Biederman et al., 2021 [45]	African American women in Gastonia, NC, USA.	Pre-test and Post-test quasi-experiment. 5-week intervention via Facebook and Pedometers. The intervention was evidence-based, but no specific model/theory was used.	20. No defined control group.	7	Female 100%	African Americans 100%	40	Hypertension
Brewer et al., 2022 [43]	African American churches in the USA.	6-month pilot cluster Randomised Control Trial (RCT). The intervention was via a web-based mobile App called FAITH. The intervention was designed according to the Precaution Adoption Process Model (PAPM).	76. Test group (n = 34). Control group (n = 42).	9	Female 71%. Male 29%.	African Americans 100%	54.5	Cardiovascular diseases
Carter et al., 2011 [46]	Washington, DC, USA.	9-month RCT focusing on weight, blood pressure, and glucose measurement using a laptop with peripherals such as a blood pressure cuff, glucometer, and wireless scale. The intervention was not based on any specific model/theory.	47. Test group (n = 26). Control group (n = 21).	27	Female 63.8%. Male 36.2%	African Americans 100%	56	Diabetes
Gerber et al., 2013 [47]	Two community churches in Chicago, IL, USA.	3-month RCT for weight loss and 9-month RCT for weight maintenance. The intervention was via video telehealth. The intervention was not based on any specific theory/model.	89. Test group (n = 45). Control group (n = 43).	5	Female 100%	African Americans 100%	50	Obesity and Overweight
Joseph et al., 2015 [48]	Metropolitan area of Phoenix, AZ, USA.	8-week, 2-arm RCT. The intervention was delivered via Facebook and text messages. The intervention was based on Social Cognitive Theory.	29. Test group (n = 14). Control group (n = 15).	0	Female 100%.	African Americans 100%	35.5	Physical inactivity
Joseph et al., 2016 [49]	College students in Phoenix, AZ, USA.	3-month, single group, pre-test and Post-test, quasi-experiment. A culturally relevant physical activity (PA) promotion website and four moderate-intensity PA sessions per week were used to deliver the intervention. The intervention was based on Social Cognitive Theory.	31. No defined control group.	6	Female 100%	African Americans 100%	21.9	Obesity and Overweight
Migneault et al., 2012 [50]	Urban-dwelling AA adults in the USA.	8-month RCT. The intervention was delivered through a Telephone Linked-Care system, an automated, computer-based, interactive telephone counselling system. The intervention was based on Social Cognitive Theory, Motivational Interviewing, and the Transtheoretical behavioral change model.	337. Test group (n = 169). Control group (n = 168).	72	Female 70% Male 30%	African American 100%	56.5	Hypertension
Pekmezi et al., 2010 [51]	Community dwellers in Rhodes Island and Pittsburgh, PA, USA.	1-year, 3-arm RCT. Intervention delivered via Tailored Internet, Tailored Print, or Standard Internet. The intervention was based on Transtheoretical Model and Social Cognitive Theory constructs.	38 Test group (n = 38). Control group (n = 211).	8	Female 92.6%. Male 7.4%	African American 100%	42.6	Obesity and Overweight
Schoenthaler et al., 2020 [52]	Primary care clinic in New York City, NY, USA.	3-month RCT. The intervention was via a mHealth device built using Microsoft’s Models, Views, and Controllers Entity Framework as the development environment. The intervention was based on the Information-Motivation-Behavioural skills model of adherence.	Phase 1 (n = 10). Phase 2 (n = 42). Test group (n = 21). Control group (n = 21).	0	Phase 1: Female 70%, Male 30%. Phase 2: Female 45.2%, Male 54.8%	African Americans 100%	Phase 1: 65.8 Phase 2: 57.6	Hypertension and Diabetes
Staffileno et al., 2018 [53]	Young African American Women in the USA.	2-arm, 3-month RCT. The intervention was web-based and accessible via the Internet and mobile devices. Intervention is designed according to Social Cognitive Theory.	35. Both control and test groups received IbI.	9	Female 100%	African Americans 100%	35.2	Pre-hypertension
Steinberg et al., 2014 [54]	Five community health centres in NC, USA.	2-arm, 12-month RCT. The intervention utilised the interactive obesity treatment approach (iOTA). Several behaviours change theories were used in the design of the intervention.	194. Test group (n = 97). Control group (n = 97).	9	Female 100%	African American 100%	35.4	Obesity
Washington-Plaskett et al., 2021 [44]	Atlanta Metro area, USA.	6-month RCT. The intervention was via Health360x, a web-based or mobile application that supports behaviour. The intervention took a theory-based approach to engage vulnerable populations in a technology-enabled behavioural intervention.	146. Both control and test groups received IbI.	26	Female 66.7%. Male 33.3%	African Americans 100%	55.6	CVDs
Webb Hooper et al., 2021 [55]	A Midwestern city in the USA.	6-week, 2-arm pilot RCT. The intervention was a video text-messaging program known as Path2Quit. The intervention was not based on any specific model/theory.	119. Intervention group (n = 61). Control group (n = 58).	9	Female 52%. Male 48%.	African Americans 100%	53.5	Smoking

**Table 4:** Summary of Study Outcomes and Quality.

Study	Purpose	Findings	Jadad Quality Rating
Biederman et al., 2021 [45]	Evaluate the efficacy of combining Facebook^TM^ and pedometers to provide a physical activity intervention to African American women.	Weekly steps increased by 190% in participants after the intervention (*p* = 0.005). Compared to baseline, about 80% of participants reported being active at least twice a week (35.7%).	Low (Jadad Score 2/5)
Brewer et al., 2022 [43]	Evaluate the feasibility and preliminary effectiveness of a web-based application promoting LS7 among African American churchgoers.	The primary outcomes are significant changes in LS7 score from baseline after six months of intervention and app engagement/usability.	High (Jadad Score 3/5)
Carter et al., 2011 [46]	Report the design, implementation, and outcomes of IbIs targeting African Americans with type 2 diabetes living in urban areas.	The results indicate that participants have favourable outcomes in decreased haemoglobin A1c and body mass index measures compared to the control group.	High (Jadad Score 3/5)
Gerber et al., 2013 [47]	Based on group interaction, assess the impact of home telehealth on weight maintenance following a weight loss programme.	Both control and test groups saw no significant changes in weight during maintenance. The groups did not have significant differences regarding nutrition, exercise, social support, or feelings of self-efficacy throughout the maintenance phase.	High (Jadad Score 3/5)
Joseph et al., 2015 [48]	Examine the effectiveness of a multi-component intervention based on the Social Cognitive Theory and utilising Facebook and text messages to encourage physical activity in African American women.	Facebook and text message-based physical activity reduced sedentary behaviour, increased light, and moderate-lifestyle intensity physical activity, improved psychosocial outcomes and increased participant satisfaction.	High (Jadad Score 4/5)
Joseph et al., 2016 [49]	Evaluate the efficacy of an Internet-enhanced physical activity (PA) pilot programme created for overweight/obese AA female college students.	This exploratory study provides early evidence in favour of IbIs being used to promote PA in overweight or obese AA women.	Low (Jadad Score 2/5)
Migneault et al., 2012 [50]	Analyse the efficacy of a culturally tailored automated phone system for hypertensive urban African-American adults and evidence-based recommendations for better eating habits and physical activity.	The intervention improved the food quality and the amount of energy expended in general. Systolic BP decreased, but the drop was not statistically significant.	High (Jadad Score 3/5)
Pekmezi et al., 2010 [51]	A subsample of AA adults was studied to see if Internet-powered, multiple contact physical activity interventions were feasible and effective.	The findings indicate that computer-tailored and Internet-based therapies can result in significant long-term gains in physical activity and associated process variables in AA adults.	High (Jadad Score 3/5)
Schoenthaler et al., 2020 [52]	Evaluate the acceptability (phase 1) and preliminary efficacy (phase 2) of a customised mobile health intervention to improve medication adherence, diastolic blood pressure (DBP), haemoglobin A1c (HbA1c), and systolic blood pressure (SBP) in black patients.	During Phase 1 semi-structured interviews, interferences in daily routines, concerns about side effects, forgetfulness, the difficulty of medication administration, and a desire for natural treatments were all identified as significant hurdles to adherence. Both groups exhIbIted considerable improvements in medication adherence and SBP in Phase 2, although there was no meaningful change.	High (Jadad Score 4/5)
Staffileno et al., 2018 [53]	Examine the effectiveness of an Internet-based, culturally relevant lifestyle change intervention for AA women to promote PA and a balanced diet.	The eHealth platform offers an alternative strategy to target young AA women and was beneficial in reducing PA and dietary behaviours.	High (Jadad Score 4/5)
Steinberg et al., 2014 [54]	Explore the patterns and predictors of low-income black women’s self-monitoring adherence to Interactive Voice Recognition (IVR) and the connection between adherence and weight change.	Adherence of socioeconomically disadvantaged black women to the IbI was high. Using IVR to encourage self-monitoring has the potential for widespread use and long-term sustainability.	High (Jadad Score 3/5)
Washington-Plaskett et al., 2021 [44]	Examine the impact of a technology-based intervention on behaviour change among AA with high cardiovascular risk in Atlanta, Georgia.	This study reveals that improvements in LS7 are associated with a 7% reduction in incident CVDs throughout a lifetime, and self-management aided by technology may be a viable way for Blacks to manage certain CVD risk factors. Females statistically significantly improved their BMI and diastolic blood pressure and decreased their self-reported physical activity. Health coaches can assist persons living in high-risk neighbourhoods in improving their overall LS7.	High (Jadad Score 3/5)
Webb Hooper et al., 2021 [55]	Determine the acceptability and short-term effects of a culturally tailored mobile health (mHealth) intervention (Path2Quit) among a sample of poor African American (AA) individuals.	It was found that a culturally-specific mHealth intervention improved Nicotine Replacement Therapy (NRT) and short-term abstinence.	High (Jadad Score 4/5)

## Data Availability

Not applicable.
